# Progress Toward Poliomyelitis Eradication — Afghanistan, January 2017–May 2018

**DOI:** 10.15585/mmwr.mm6730a6

**Published:** 2018-08-03

**Authors:** Maureen Martinez, Hemant Shukla, Meiland Ahmadi, Joanna Inulin, Mufti Sabari Widodo, Jamal Ahmed, Chukwuma Mbaeyi, Jaime Jabra, Derek Gerhardt

**Affiliations:** ^1^Global Immunization Division, Center for Global Health, CDC; ^2^Polio Eradication Department, World Health Organization, Geneva, Switzerland; ^3^Polio Emergency Operations Center, Ministry of Public Health, Kabul, Afghanistan; ^4^Division of Viral Diseases, National Center for Immunization and Respiratory Diseases, CDC.

Afghanistan, Pakistan, and Nigeria remain the only countries where transmission of endemic wild poliovirus type 1 (WPV1) continues (*1*). This report describes polio eradication activities, progress, and challenges to eradication in Afghanistan during January 2017–May 2018 and updates previous reports (*2*, *3*). Fourteen WPV1 cases were confirmed in Afghanistan in 2017, compared with 13 in 2016; during January–May 2018, eight WPV1 cases were reported, twice the number reported during January–May 2017. To supplement surveillance for acute flaccid paralysis (AFP) and laboratory testing of stool samples, environmental surveillance (testing of sewage samples) was initiated in 2013 and includes 20 sites, 15 of which have detected WPV1 circulation. The number of polio-affected districts increased from six in 2016 to 14 in 2017 (including WPV1 cases and positive environmental samples). Access to children for supplementary immunization activities (SIAs) (mass campaigns targeting children aged <5 years with oral poliovirus vaccine [OPV], regardless of vaccination history), which improved during 2016 to early 2018, worsened in May 2018 in security-challenged areas of the southern and eastern regions. To achieve WPV1 eradication, measures to maintain and regain access for SIAs in security-challenged areas, strengthen oversight of SIAs in accessible areas to reduce the number of missed children, and coordinate with authorities in Pakistan to track and vaccinate mobile populations at high risk in their shared transit corridors must continue.

## Immunization Activities

The World Health Organization (WHO) and UNICEF estimated national routine vaccination coverage of infants aged <12 months with 3 doses of OPV (OPV3) in Afghanistan at 60% in both 2015 and 2016 (*4*). As a proxy indicator for national OPV3 coverage, the percentage of children aged 6–23 months with nonpolio acute flaccid paralysis (NPAFP) who received 3 OPV doses through routine immunization services was 67% in 2016 and 68% in 2017. Administrative OPV3 coverage (calculated by dividing the number of doses administered by the estimated target population) in 2017 ranged from 100% in the central provinces of Kapitsa and Panjsher to 24% and 9% in the southern provinces of Helmand and Kabul, respectively. The proportion of children aged 6–23 months nationally with NPAFP who never received OPV through routine immunization services or SIAs (i.e., “zero-dose” children) was approximately 1% during 2016–2017. High proportions of zero-dose children were reported in 2017 in Kabul (9%) and Kandahar (4%) provinces in the southern region, Kunar (8%) province in the eastern region, and Paktika (7%) province in the southeastern region.

During January 2017–May 2018, SIAs targeted children aged <5 years for receipt of monovalent OPV (containing only type 1 vaccine virus) or bivalent OPV (containing types 1 and 3 vaccine viruses) including six national immunization days (NIDs), nine subnational immunization days and one “mop-up” SIA (door-to-door immunization campaigns carried out in specific areas where the virus is known or suspected to still be circulating). Injectable inactivated poliovirus vaccine (IPV) was administered during SIAs to 1,248,749 children aged 4–59 months who lived in districts designated as very high-risk for polio circulation or in areas that had been inaccessible for previous SIAs to help boost their immunity to the virus and decrease the risk for paralytic disease.

During the period covered by this report, NIDs targeted 9,999,227 children aged <5 years. Children missed for vaccination during NIDs are recorded either as inaccessible* or as accessible but missed because of campaign quality issues including vaccine refusal; team failure to reach the home; or child being sick, asleep, or absent. During the September 2017 NIDs, a total of 362,276 children (3.6%) were reported as having been missed, including 152,201 (1.5%) reported as inaccessible and 210,075 (2.1%) as accessible. During the March 2018 NID, the number of missed children was reduced to 290,510 (2.9%) with 101,561 (1.1%) inaccessible and 188,949 (1.8%) accessible. In May 2018 NIDs, the number of inaccessible children increased to 996,326 (9.9%) with the greatest numbers missed in Helmand (76.2% of all targeted children) and Urozgan (100% of targeted) provinces in the southern region because of a ban on vaccination. During this round, 204,354 (2.0%) children also were missed in accessible areas.

Lot quality assurance sampling^†^ surveys are used to assess the quality of SIAs in accessible areas. The percentage of districts that reportedly failed at the 80% threshold was 16.7% during the September 2017 NID, 7.1% during the March 2018 NID, and 8.3% during the May 2018 NID, which indicates improved campaign quality during 2017–2018 to date.

Children aged <5 years also are targeted for vaccination along major travel routes throughout the country, at transit points from inaccessible areas, and at border crossing points with Pakistan and Iran (which target children aged <10 years). Approximately 13 million OPV doses were administered at transit points during 2017, and approximately 5.7 million doses were administered during January–May 2018. At border crossings, approximately 830,000 children were vaccinated during 2017, and approximately 340,000 during 2018 to date.

## Poliovirus Surveillance

**Acute flaccid paralysis surveillance**. Surveillance for AFP, which is a highly sensitive surveillance system to detect a case of polio, is a critical component of the global polio eradication initiative; the target is ≥2 NPAFP cases per 100,000 persons aged <15 years (*5*). The surveillance network includes government and private health facilities, shrines, traditional healers, and approximately 35,000 reporting volunteers. In 2017, the annual national NPAFP rate was 15.3 per 100,000 persons aged <15 years (provincial range, 11.4–20.4) (Table). The percentage of AFP cases with adequate stool specimens^§^ collected was 93.5% (range, 87.5%–96.9%); the target is ≥80% of AFP cases. During January 2017–May 2018, the NPAFP rate exceeded 12 per 100,000 persons aged <15 years, and the percentage of AFP cases with adequate stool specimens exceeded 91% across all SIA access categories.

**TABLE:** Acute flaccid paralysis (AFP) surveillance indicators and reported cases of wild poliovirus (WPV), by region and period — Afghanistan, January 2017–May 2018*

Region of Afghanistan	AFP surveillance indicators (2017)			No. of WPV cases reported		
	No. of AFP cases	Rate of nonpolio AFP^†^	% of AFP cases with adequate specimens^§^	Jan–Jun 2017	Jul–Dec 2017	Jan–May 2018
**All regions**	**3,078**	**15.3**	**93.5**	**5**	**9**	**8**
Badakhshan	65	11.4	95.4	0	0	0
Northern	345	13.7	92.8	0	0	0
Northeastern	421	18.7	91.9	1	0	0
Central	545	11.8	96.9	0	0	0
Eastern	363	18.3	93.9	0	3	3
Southeastern	250	12.7	94.8	0	0	0
Southern	543	15.4	87.5	4	6	5
Western	546	20.4	95.1	0	0	0

* Data current through May 15, 2018.

^†^ Per 100,000 persons aged <15 years. Surveillance target is ≥2/100,000 persons aged <15 years.

^§^ Surveillance target is that ≥80% of AFP cases have adequate stool specimens collected. Adequate stool specimens are defined as two stool specimens of sufficient quality for laboratory analysis, collected ≥24 hours apart, both within 14 days of paralysis onset, and arriving in good condition at a World Health Organization–accredited laboratory with reverse cold chain maintained and without leakage or desiccation and with proper documentation.

**Environmental surveillance.** Supplementary poliovirus surveillance in Afghanistan is conducted through sampling of sewage at 20 sites in nine provinces. Sampling frequency from eight sites in the southern region has been increased from monthly in 2016 to every 2 weeks since 2017. WPV1 was detected in two (1%) of 184 specimens tested in 2016, 42 (13%) of 316 specimens tested in 2017, and 21 (16%) of 148 specimens tested in 2018 to date (from Nangarhar, Kunar, Kandahar, Helmand, and Kabul provinces).

## Epidemiology of WPV Cases

**FIGURE 1:**
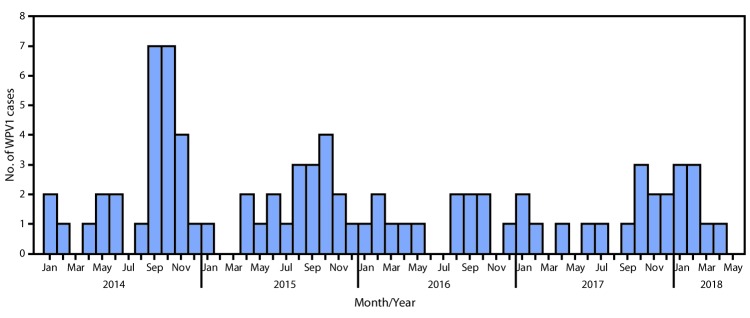
Number of wild poliovirus type 1 (WPV1) cases (n = 83) — Afghanistan, January 2014–May 2018

**FIGURE 2:**
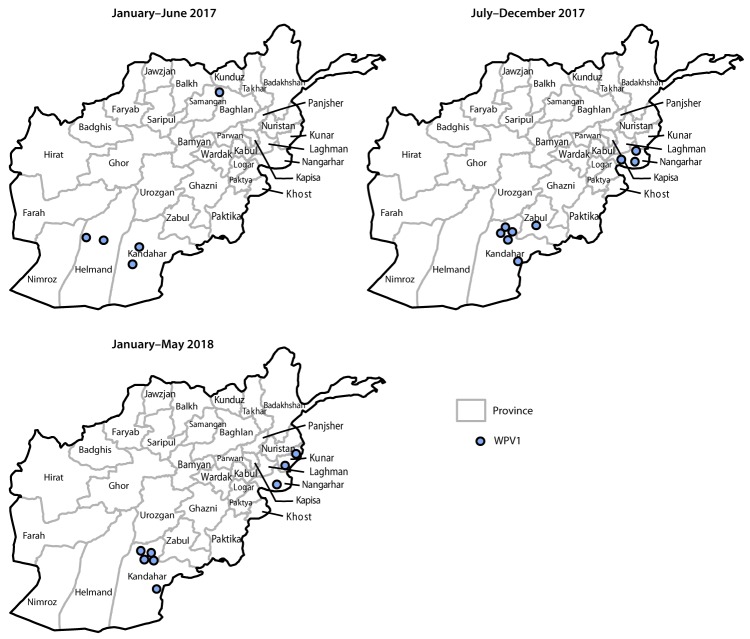
Cases of wild poliovirus type 1 (WPV1),* by province — Afghanistan, January 2017–May 2018 * Each dot represents one case. Dots are randomly placed within provinces.

During 2017, 14 WPV1 cases were reported in five provinces (Kandahar [seven cases], Nangarhar [three], Helmand [two], Zabul [one], and Kunduz [one]) compared with 13 in four provinces in 2016. Eight WPV1 cases were reported during January–May 2018, compared with four during January–May 2017 (Figure 1) (Figure 2). The number of districts reporting WPV1 cases increased from four in 2016 to nine in 2017. As of June 29, 2018, eight cases had been reported, including five from Kandahar, two from Kunar, and one from Nangarhar provinces. Among the 22 cases reported during January 2017–May 2018, 17 (77%) were among children aged <36 months, nine of 22 (41%) had never received OPV by any means (i.e., routine immunization or SIA), two (9%) had received 1 or 2 doses, and 11 (50%) had received ≥3 doses each; 15 (68%) of the 22 children with polio had never received OPV through routine immunization services.

Genomic sequence analysis of poliovirus isolates identified multiple episodes of cross-border transmission during 2017–2018 between Pakistan and Afghanistan, with sustained local transmission in both countries. During January 2017–May 2018, 11 (50%) of 22 isolates from patients with AFP and 35 (57%) of 62 isolates from environmental testing identified in Afghanistan had closest genetic links to earlier WPV1 isolates from Pakistan; the remaining isolates were most closely linked to isolates identified in Nangarhar, Kabul, Kandahar, and Helmand provinces of Afghanistan. During 2018, two genetic clusters (viruses sharing ≥95% of sequence identity) were detected from AFP cases; one cluster of five cases occurred in the southern province of Kandahar and the other three cases occurred in two adjacent provinces (Nangarhar and Kunar) in eastern Afghanistan. Whereas the number of yearly cases has been fairly steady from 2016 to 2018 to date, the geographic location of WPV cases in 2018 has been restricted to the historically shared reservoirs of poliovirus transmission between Afghanistan and Pakistan, known as the Northern and Southern Corridors.^¶^

## Discussion

During the period covered by this report, the number of WPV1 cases and positive environmental isolates in Afghanistan has increased modestly each year since 2016. However, the geographic extent of virus circulation appears limited to the southern and eastern regions. Despite security challenges, the major surveillance performance indicators are being met across all levels of access. Critical issues that need to be addressed to interrupt WPV1 circulation are reaching children for vaccination in inaccessible areas, reaching a higher proportion of children in accessible areas, and coordinating vaccination programs with Pakistan to better reach mobile populations at high risk that travel between the two countries.

Inaccessibility continues to be a barrier to reaching children during SIAs, particularly in the eastern and southern regions. The primary strategy to address access has been to engage in dialogue with local influencers and antigovernment elements. This strategy has been successful in reducing the number of inaccessible areas; however, access has become increasingly challenging in recent months because the number of bombings, attacks, active conflicts, and campaign bans have increased. In the face of mounting insecurity, the program must continue to identify ways to gain and maintain access to children in security-compromised areas.

The quality of campaigns in accessible areas has improved, as evidenced by lot quality assurance sampling surveys and postcampaign coverage data. To improve SIA quality in accessible areas, religious leaders and prominent community members are asked to identify children missed in SIAs and to dispel misperceptions about vaccination. A national exercise is underway to update all microplans throughout the country to ensure the house locations for all eligible children are identified, so that they are included in each NID. Measures to vaccinate accessible children missed during SIAs because of poor campaign quality need to be enhanced, particularly regarding vaccine refusals.

During 2017−2018, the polio program in Afghanistan identified 28 districts at highest risk for poliovirus circulation and developed action plans in coordination with the Pakistan polio program for their shared high-risk areas in the Northern and Southern Corridors. Cross-border coordination with Pakistan continues to be enhanced to identify, track, and vaccinate mobile populations at high risk. Both countries have synchronized their vaccination campaign schedules to ensure that mobile populations are not missed. The number of permanent transit teams targeting mobile populations at high risk and children exiting inaccessible areas continues to be increased.

To interrupt WPV transmission, the country program must continue to identify innovative ways to access children in security-compromised areas and enhance measures to vaccinate accessible children missed during SIAs and among mobile populations at high risk. The country program also must cooperate closely with the polio program in Pakistan.

**Summary**

**What is already known about this topic?**

Afghanistan is one of three countries where transmission of wild poliovirus continues.

**What is added by this report?**

The annual number of reported wild poliovirus cases in Afghanistan has remained steady since 2016 although the geographic spread has decreased. Access to children for immunization campaigns improved during 2017 through early 2018 but worsened in May 2018 in security-challenged areas of the southern and eastern regions.

**What are the implications for public health practice?**

To interrupt wild poliovirus transmission in Afghanistan, progress is needed toward regaining campaign access in security-challenged areas, improving campaign quality in accessible areas, and enhancing coordination with Pakistan to track and vaccinate mobile populations at high risk.

## Footnotes

* SIA access at the district level is divided into four categories: not accessible, partially accessible, accessible with limitation, and fully accessible. Partially accessible indicates that the campaign was implemented fully but only in part of the district, whereas implemented with limitation indicates that the campaign was implemented throughout the entire district but with restrictions to monitoring.

† Lot quality assurance sampling is a rapid survey method used to assess the quality of vaccination activities after SIAs in predefined areas, such as health districts (referred to as “lots”), using a small sample size. Lot quality assurance sampling involves dividing the population into lots and randomly selecting persons in each lot. If the number of unvaccinated persons in the sample exceeds a predetermined value, then the lot is classified as having an unsatisfactory level of vaccination coverage, and mop-up activities are recommended. If the threshold of ≥80% is met, the area or district is classified as having passed, although mop-up activities might still be indicated in certain areas.

§ At least 80% of AFP cases should have adequate stool specimens collected. Adequate stool specimens are defined as two stool specimens of sufficient quantity for laboratory analysis, collected ≥24 hours apart, both within 14 days of paralysis onset, and arriving in good condition at a WHO-accredited laboratory with reverse cold chain maintained and without leakage or desiccation and with proper documentation.

¶ The Northern Corridor includes the provinces of Nangarhar, Kunar, Laghman, and Nuristan in Afghanistan and Bajour agency, Mohmand agency, Khyber agency, and FR Peshawar in the Northern Federally Administered Tribal Areas and the districts of Peshawar, Nowshera, and Charsadda, Mardan, Swabi, Swat, Malakand, Dir Lower, Dir Upper, and Chitral Khyber Pakhtunkhwa in Peshawar in Pakistan. The Southern Corridor includes Kandahar and Helmand provinces in Afghanistan and the districts of Quetta, Killa Abdullah, and Pishin in Baluchistan province of Pakistan.
