# *Erratum: *Vol. 67, No. 30

**DOI:** 10.15585/mmwr.mm6732a7

**Published:** 2018-08-17

In the report “Progress Toward Poliomyelitis Eradication — Afghanistan, January 2017–May 2018,” multiple incorrect spellings occurred because of a spell-check error. The errors are corrected online.

The listing of authors originally read “Maureen Martinez, MPH^1^; Hemant Shukla, MD^2^; Meiland Ahmadi, MD^3^; Joanna Inulin, MD^2^; Mufti Sabari Widodo, MBBS^2^; Jamal Ahmed, MD^2^; Chukwuma Mbaeyi, DDS^1^; Jaime Jabra, PhD^4^; Derek Gerhardt, MPH^1^”

The listing of authors should have read “Maureen Martinez, MPH^1^; Hemant Shukla, MD^2^; **Maiwand Ahmadzai**, MD^3^; **Joanna Nikulin**, MD^2^; **Mufti Zubair Wadood**, MBBS^2^; Jamal Ahmed, MD^2^; Chukwuma Mbaeyi, DDS^1^; **Jaume Jorba**, PhD^4^; **Derek Ehrhardt**, MPH^1^”

The first paragraph of text under Immunization Activities on page 833 beginning with the third sentence originally read “Administrative OPV3 coverage (calculated by dividing the number of doses administered by the estimated target population) in 2017 ranged from 100% in the central provinces of Kapitsa and Panjsher to 24% and 9% in the southern provinces of Helmand and Kabul, respectively. The proportion of children aged 6–23 months nationally with NPAFP who never received OPV through routine immunization services or SIAs (i.e., “zero-dose” children) was approximately 1% during 2016–2017. High proportions of zero-dose children were reported in 2017 in Kabul (9%) and Kandahar (4%) provinces in the southern region, Kunar (8%) province in the eastern region, and Paktika (7%) province in the southeastern region.”

The sentences should have read “Administrative OPV3 coverage (calculated by dividing the number of doses administered by the estimated target population) in 2017 ranged from 100% in the central provinces of **Kapisa** and Panjsher to 24% and 9% in the southern provinces of Helmand and **Zabul**, respectively. The proportion of children aged 6–23 months nationally with NPAFP who never received OPV through routine immunization services or SIAs (i.e., “zero-dose” children) was approximately 1% during 2016–2017. High proportions of zero-dose children were reported in 2017 in **Zabul** (9%) and Kandahar (4%) provinces in the southern region, Kunar (8%) province in the eastern region, and Paktika (7%) province in the southeastern region.”

